# Two Cases of Lung Injury During the Nuss Procedure for Pectus Excavatum

**DOI:** 10.7759/cureus.85952

**Published:** 2025-06-13

**Authors:** Kazuki Sato, Masahiro Miyajima, Taiki Sato, Yuma Shindo, Atsushi Watanabe

**Affiliations:** 1 Department of Thoracic Surgery, Sapporo Medical University, Sapporo, JPN

**Keywords:** complications, lung injury, nuss procedure, pectus excavatum, thoracic surgery

## Abstract

The Nuss procedure is a minimally invasive surgery commonly performed for pectus excavatum. However, it can cause fatal complications during surgery, making it crucial to prevent these complications. Here, we report two cases of lung injury during the procedure and discuss their causes and preventive measures. Two male patients, aged 18 and 19 years, with a Haller index of 5.9 and 3.5, respectively, underwent the Nuss procedure under thoracoscopic guidance. In both cases, the introducer tip penetrated the left lingular segment during passage, with an injury being discovered upon bar insertion. The lung injuries were repaired thoracoscopically using sutures, with uneventful postoperative recovery. Although the Nuss procedure is minimally invasive, it carries risks, including lung injury. Our experience highlights the importance of visualizing the introducer tip during the passage to avoid complications. We recommend a 4-5 cm wide mediastinal pleural incision, thoracoscopic confirmation of introducer placement, and addition of a left thoracic port for improved visualization. Both the surgeon's experience and the age of the patient may contribute to complications. These cases highlight the importance of meticulous technique and vigilant execution during the Nuss procedure.

## Introduction

The Nuss procedure has been widely performed as a standard surgical treatment for pectus excavatum since it was first reported by Donald Nuss in 1998 [1]. It is a minimally invasive procedure, involves small incisions and does not require osteochondrotomies. However, there have been reports of serious complications, and fatalities have also been observed [2,3]. Complications during the Nuss procedure include life-threatening events such as cardiac perforation by the clamp or pectus bar, though these have rarely occurred. Moss et al. reported a case of cardiac perforation with the clamp passing through the right atrium and the right ventricle. The surgeons promptly made a midline sternotomy, initiated a cardiopulmonary bypass, and repaired the cardiac injury [2]. Gips et al. reported a case of cardiac perforation with the pectus bar penetrating the anterior aspect of the heart, leading to death [4]. Bilgi et al. reported 15 cases of lung parenchymal laceration, which occurred during the blind insertion of the trocar or the dividing lung adhesions in the Nuss procedure [5]. Kim et al. reported a case of lung entrapment between the pectus bar and chest wall after the Nuss procedure. They found the entrapment of the right middle lobe when performing thoracoscopic surgery to treat pneumothorax with a persistent collapsed lung, which developed on the fourth postoperative day. They thought that the damaged lung might be related to the air leak [6]. Henry et al. reported a case of lung laceration from adhesions between the pleura and the pectus bar, which occurred during the removal of the bar and required lobectomy to control bleeding [7]. According to literature, the overall complication rate is 4.98%, and the most common complication reported is pneumothorax, ranging from 1-4%, while lung injury is reported in 2.3% of cases [5,8]. Among the complications, lung injury requires additional surgical repair, highlighting the importance of prevention. We present two cases of lung penetration during the Nuss procedure.

## Case presentation

Ethical approval

Written informed consent was obtained from both patients.

Venue of surgical correction

The surgical corrections for these cases were performed at the Sapporo Medical University, School of Medicine, Sapporo, Japan.

Case 1

**Figure 1:**
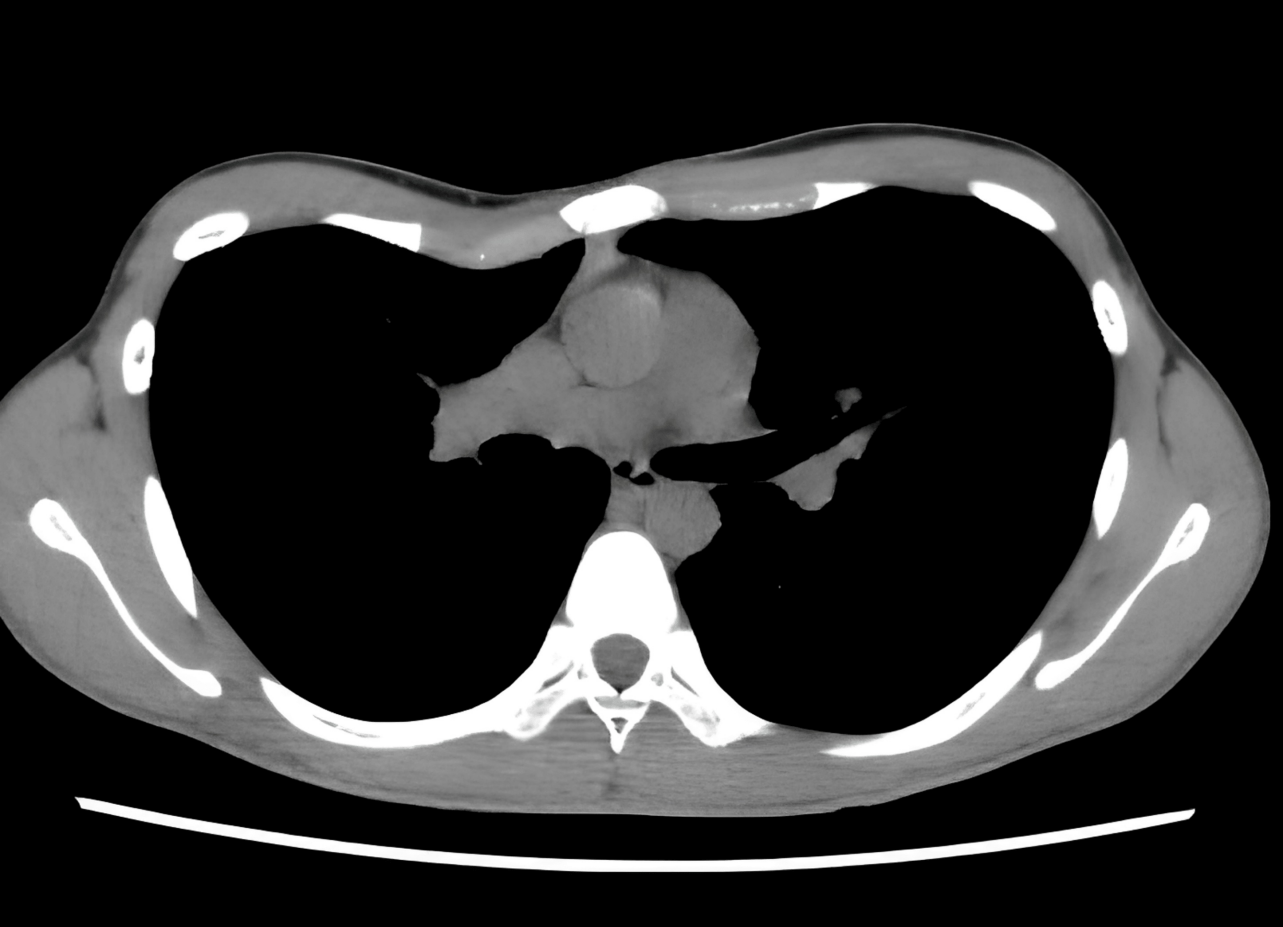
Chest computed tomography (CT) images of Case 1 (Haller index=3.5) Asymmetrical depression is observed in the sternum, predominantly on the right side of the midline.

An 18-year-old male patient was diagnosed with pectus excavatum during a junior high school health screening. He was under observation due to the absence of symptoms; however, after turning 18, he developed exertional dyspnea. The patient was referred to our department for surgery. Preoperative chest computed tomography (CT) revealed asymmetrical sternal depression, more to the right, as shown in Figure 1.

The Haller index was 3.5 [9], and the pulmonary function test was within normal limits (Table 1).

**Table 1:** The results of pulmonary function tests (Case 1) Pulmonary function tests were within normal limits. VC: Vital Capacity; FEV1.0: Forced Expiratory Volume in one second; FVC: Forced Vital Capacity

Parameter	Result	Reference range
VC (L)	3.57	3.0–5.0 L
%VC (%)	84.8	≥80%
FEV1.0 (L)	3.46	≥2.0 L
FEV1.0% (%)	95.1	≥70%
FVC (L)	3.64	3.5–5.5 L
%FVC (%)	86.5	≥80%

Surgical Procedure

**Figure 2:**
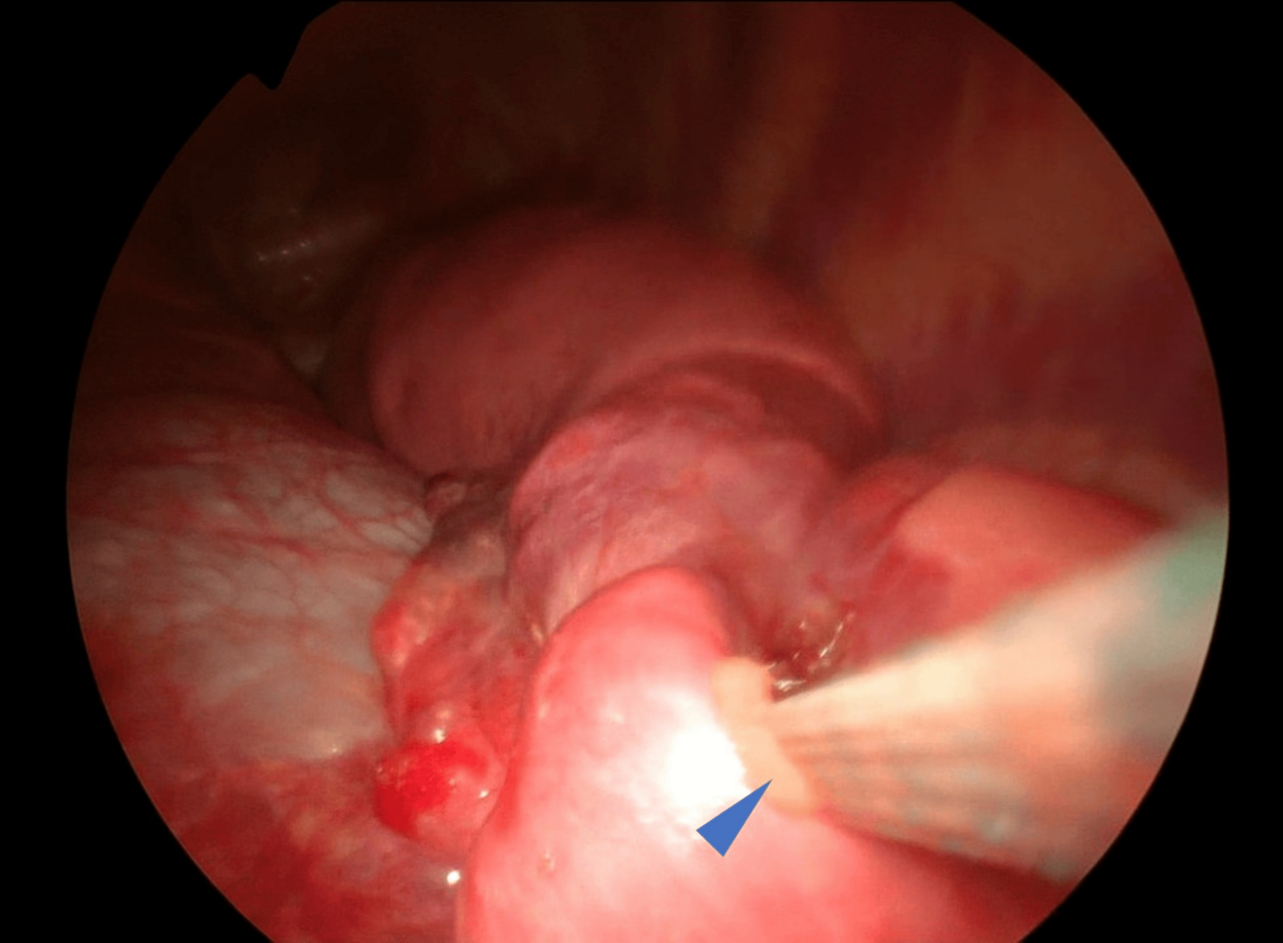
The vascular tape has penetrated the lingular segment of the lung (Case 1) The blue arrowhead indicates the portion where the vascular tape passed from the right pleural cavity to the left pleural cavity, traversing the lingular segment of the left lung.

Under general anesthesia with one-lung ventilation, the patient was placed in the supine position. We marked the deepest point of the sternal depression. The bar was planned for placement in the intercostal space at the highest point of both lateral chest walls, in line with this sternal mark. Skin incisions were planned at the intersection of this extended line and the anterior axillary line. Skin incision markings were placed at the intersection of a horizontal extension from the highest point and the mid-axillary line, where 3 cm horizontal incisions were then made. A 5-mm-diameter thoracoscope (Olympus Corporation, Tokyo, Japan) was inserted into the right thoracic cavity through the seventh intercostal space. Bilateral incisions were made along the marked lines. The subcutaneous tissue was dissected around the incisions and then separated upward to the highest points of the chest wall marked bilaterally. The introducer (Pectus Introducer, Zimmer Biomet Microfixation, Florida, USA) was inserted into the right thoracic cavity from the highest point on the right chest wall. Under thoracoscopic guidance, the posterior sternal tissue was separated, and the introducer was withdrawn from the highest point on the left chest wall. Two vascular tape strips were tied to the introducer tip, and the guide tape was placed into the left thoracic cavity through the highest point of the right chest wall. A shaped bar (Pectus Bar, Zimmer Biomet Microfixation, Florida, USA)was then tied to the vascular tape and, guided by the tape, inserted from the right thoracic cavity to exit at the highest point of the left thoracic cavity. However, resistance prevented full penetration of the chest wall, requiring temporary bar removal. Thoracoscopic observation from the left incision revealed that the vascular tape had penetrated the left lingular segment, damaging the introducer (Figure 2).

The vascular tape was removed, and a 5-mm port was added to the left seventh intercostal space at the anterior axillary line. Lung repair was performed using two 2-0 polydioxanone sutures (PDS; Johnson & Johnson, New Jersey, USA) with a fascia patch. A water-seal leak test confirmed no air leakage. The bar was reinserted from the right thoracic cavity, passed through the left thoracic cavity, and exited through the left chest wall using a vascular tape guide. The bar was rotated 180° with a rotator to elevate the chest wall. The fifth rib and bar were fixed with 1 mm-PDS, and the bar ends were secured to the chest wall with 1-0 PDS. A left thoracic cavity 14Fr soft catheter was placed, completing the procedure. The operative time was 159 minutes, and blood loss was minimal.

The postoperative course was uneventful. The chest tube was removed on the first postoperative day, and the patient was discharged on day nine.

Case 2

**Figure 3:**
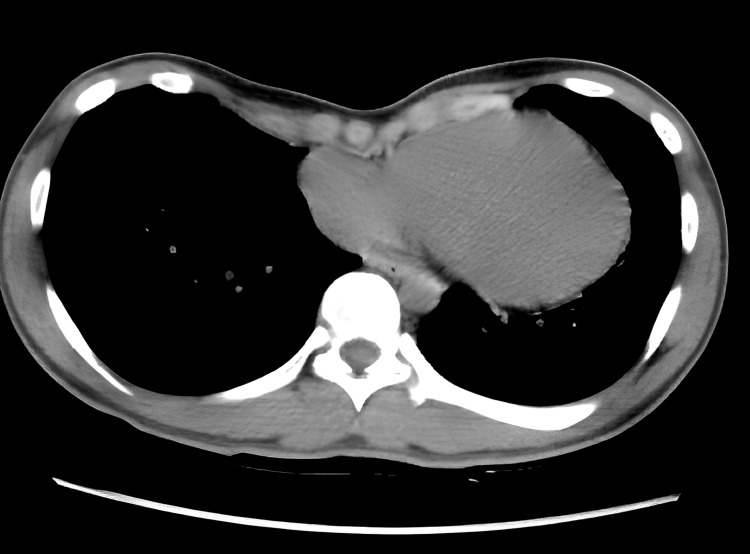
Chest CT image of Case 2 (Haller index=5.9) Symmetrical depression of the sternum and mild cardiac compression are observed.

A 19-year-old male patient, diagnosed with pectus excavatum at a nearby pediatric clinic, was referred to our department for surgical treatment. Preoperative chest CT revealed symmetrical sternal depression as shown in Figure 3.

The Haller index was 5.9, and the pulmonary function tests were within normal limits (Table 2).

**Table 2:** The results of pulmonary function tests (Case 2) Pulmonary function tests were within normal limits. VC: Vital Capacity; FEV1.0: Forced Expiratory Volume in one second; FVC: Forced Vital Capacity

Parameter	Result	Reference range
VC (L)	3.92	3.0–5.0 L
%VC (%)	88.9	≥80%
FEV1.0 (L)	3.46	≥2.0 L
FEV1.0% (%)	88.5	≥70%
FVC (L)	3.91	3.5–5.5 L
%FVC (%)	88.7	≥80%

Surgical Procedure

**Figure 4:**
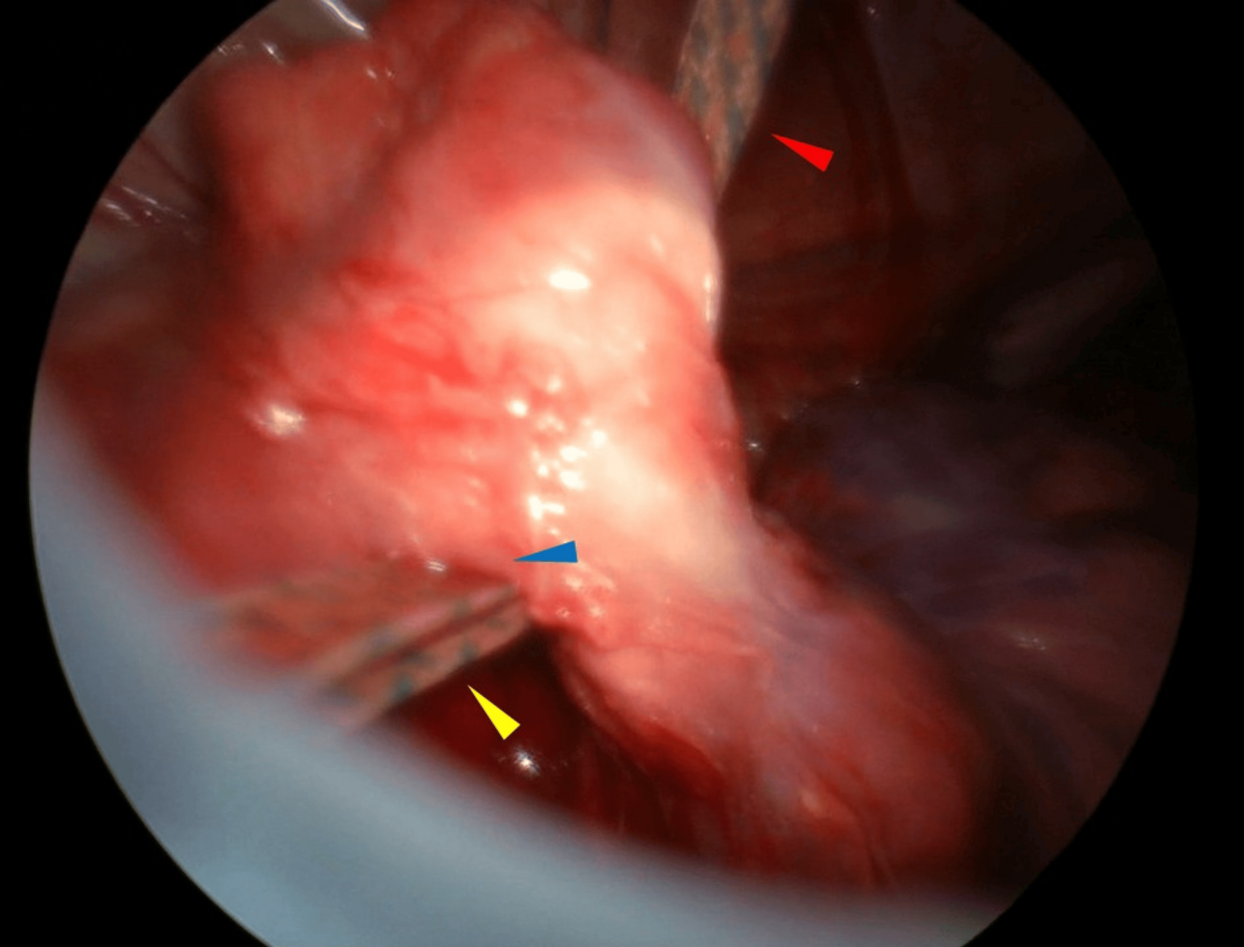
The vascular tape has penetrated the lingular segment of the lung (Case 2) The red arrowhead indicates the vascular tape that has passed from the right pleural cavity through the anterior mediastinum. The blue arrowhead indicates the portion where the vascular tape, passed from the right pleural cavity to the left pleural cavity, traverses the lingular segment of the left lung. The yellow arrowhead indicates the vascular tape that has penetrated the lingular segment of the left lung and is exiting into the left pleural cavity.

Skin incision and introducer insertion followed the same procedure as in Case 1. Vascular tape was passed from the left to the right thoracic cavity. Upon inserting the bar from the right thoracic cavity to exit the left thoracic cavity, the lung was observed through the left incision. Thoracoscopic observation via the left incision of the thoracic cavity revealed that the vascular tape had penetrated the left lingular segment (Figure 4).

A 5-mm port was inserted into the left seventh intercostal space, and the damaged left lingular segment was repaired with 4-0 PDS sutures on both the parietal and mediastinal aspects of the lung. A water-seal leak test confirmed no air leakage. A pectus bar (Pectus Bar, Zimmer Biomet Microfixation, Jacksonville, USA) was then placed in the fourth intercostal space using the same technique as in case 1, and a left 12Fr aspiration catheter (Trocar Aspiration Kit, Cardinal Health, Dublin, USA) was placed to complete the procedure. The operative time was 122 minutes, and blood loss was minimal.

The postoperative course was uneventful. The chest tube was removed on the first postoperative day, and the patient was discharged on the ninth day.

## Discussion

Donald Nuss first introduced the minimally-invasive repair of pectus excavatum in 1987. Since publishing his 10-year results in 1998, the Nuss procedure has been increasingly adopted owing to its minimally-invasive approach and superior cosmetic outcomes compared to traditional techniques [1]. In our department, the Nuss procedure is performed on patients diagnosed with pectus excavatum who meet at least two of the criteria listed in Table 3.

**Table 3:** Patient selection criteria for the Nuss surgery

Criterion	Description	Details
(a) Symptoms	Patient reports physical complaints such as shortness of breath during exertion, decreased endurance, and chest pain. A history of deformity progression, especially during puberty, is often present.	Based on medical history
(b) Physical examination	Moderate-to-severe pectus excavatum deformity is observed, which may be either symmetric or asymmetric.	Findings from physical examination
(c) Chest imaging	Severe deformity indicated by one or more of the following: Haller Index >3.2; Correction Index >10%; Evidence of cardiac and/or pulmonary compression or displacement	Evaluated by CT or MRI
(d) Pulmonary function	Restrictive or obstructive ventilatory patterns are detected.	Pulmonary function test findings
(e) Cardiac evaluation	Cardiac compression or displacement, arrhythmia, or mitral valve prolapse is identified.	Assessed via echocardiography or ECG
(f) Psychosocial impact	Significant concerns regarding body image or evidence of psychosocial maladjustment.	Based on psychological assessment or patient interview

However, fatal complications were reported. The incidence of intraoperative lung injury is unclear, but reported rates are 2-3% [2,3]. Mennie et al. reported that thoracoscopy significantly reduced intra- and postoperative complications [10].

In our department, the Nuss procedure always involves thoracoscopy. A 5-mm port is inserted through the right lower intercostal space, and scope-guided dissection is performed directly under the sternum. However, the left lung and contralateral chest wall are not visualized during introducer withdrawal from the contralateral thoracic cavity, posing a risk of damage to the left lingular segment. In both cases reported here, lung penetration occurred when the bar passed through the vascular tape and exited on the opposite side, and not during introducer insertion. The introducer's sharp tip potentially increases the risk of lung injury. To prevent this, we recommend incising the mediastinal pleura and thoracoscopically verifying that the introducer passes over the left lingular segment. Additionally, in cases with severe sternal depression, a lifting hook may improve visualization of the contralateral thoracic cavity. If visualization is difficult, a port is added to the left thoracic cavity for thoracoscopic guidance.

Based on their review of case reports and their experience, Hebra et al. deduced that the majority of life-threatening complications occurred in teenage patients over 16 years of age or adult patients [11]; our patients were 18 and 19 years old, respectively. Furthermore, complications were more commonly reported in the early series, which included the learning curves for many surgeons [12,13]. The surgeons in these two cases had less than five cases of experience and were beginners, which is also a factor in lung injury.

Repair is necessary when lung injury occurs. In this study, we added a camera port to the left seventh intercostal space and repaired the injury. Suturing or partial resection using a stapler can be performed as a repair method. In both cases, repair was performed by suturing, and no leaks were observed in the leak test. After surgery, a drain was inserted, and it was confirmed that pneumothorax did not occur later, and it was removed a few days postoperatively.

New approaches are emerging, such as Klobe's vacuum bell, which is now a treatment option for younger patients with mild-to-moderate deformities and a soft, malleable chest wall [14,15]. Previously, the Ravitch procedure was the only available technique for patients with pectus excavatum. However, a spectrum of techniques now exists, ranging from the non-operative vacuum bell treatment for mild and moderate cases, the Nuss procedure for more severe cases, to modified open procedures for very severe and recurrent cases, as well as combined open and closed procedures [16,17].

Other novel ideas are currently in the experimental stage, including Bardaji's Pectus Up procedure, which uses a screw to elevate the sternum, and Harrison's Magnetic Mini-Mover Procedure, which employs magnets to pull the sternum up over a two-year period [18].

Beyond the well-established Nuss procedure, less invasive treatment modalities for pectus excavatum are thus emerging. This evolution suggests that the management of pectus excavatum should not be solely surgical. Instead, it is crucial to carefully evaluate each case and consider conservative treatment options where appropriate.

## Conclusions

We reported two cases of lung injury during the Nuss procedure. In both cases, failure to visualize the left lung and contralateral chest wall during the introducer passage resulted in injury to the left lingular segment. Additionally, the fact that surgeons with fewer than five cases of experience performed the procedure on patients aged 16 years or older, who are prone to more complications, may have also contributed to the outcome. To prevent lung injury, it is important to visualize the introducer tip during bar insertion using all available methods. When lung injury is suspected, it's crucial to immediately place a camera port on the contralateral side for confirmation.

Surgeon experience and patient age may contribute to complications. Therefore, careful case selection should be considered during the early stages of a surgeon's career. To prevent lung injury, less experienced surgeons should ensure clear visualization of the contralateral lung when inserting the introducer.
